# A case report of H‐syndrome from Baghdad Medical City treated with tocilizumab

**DOI:** 10.1002/ccr3.6775

**Published:** 2022-12-19

**Authors:** Nabaa Ihsan Awadh, Faiq I. Gorial, Hashim Talib Hashim, Ahmed Dheyaa Al‐Obaidi, Mustafa Najah Al‐Obaidi, Ali H. AlNoori, Wesal K. Aljanabi

**Affiliations:** ^1^ Rheumatology Unit, Internal Medicine Department Baghdad Teaching Hospital, Medical City Complex Baghdad Iraq; ^2^ Rheumatology Unit, Department of Medicine, College of Medicine University of Baghdad Baghdad Iraq; ^3^ College of Medicine University of Baghdad Baghdad Iraq; ^4^ Dermatology Center, Medical City Baghdad Iraq

**Keywords:** clubbing, deafness, deformities, H syndrome, hyperpigmentation, tocilizumab

## Abstract

This case report presents the first H‐syndrome rarity in Iraq, a 12‐year‐old female patient who was attending the Rheumatology out clinic for progressive hands joint deformities. She has a history of a multi‐systemic collection of diseases with various clinical features that include beta thalassemia minor, sensorineural deafness, and celiac disease.

## Graphical abstract

H syndrome, also known as histiocytosis‐lymphadenopathy plus syndrome or PHID, is a rare genetic condition caused by mutations in the SLC29A3 gene. This case report presents the first H‐syndrome rarity in Iraq.

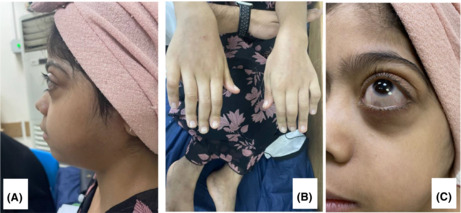

## 1 INTRODUCTION

H syndrome, also known as histiocytosis‐lymphadenopathy plus syndrome or PHID, is a rare genetic condition caused by mutations in the SLC29A3 gene which encodes the human equilibrative nucleoside transporter (hENT3) protein.^1^

It is also known as Faisalabad histocytosis, familial Rosai‐Dorfman disease, sinus histocytosis with massive lymphadenopathy and pigmented hypertrichosis with insulin‐dependent diabetes mellitus syndrome.^2^

It is a rare autosomal recessive syndrome characterized by a constellation of clinical features and systemic manifestations, including cutaneous hyperpigmentation, hypertrichosis, hepatosplenomegaly, hearing loss, heart anomalies, hypogonadism, hyperglycemia, low height, and hallux valgus. We report a case of this syndrome with typical clinical findings. We report this case citing the rarity of this uncommon entity.^1^

H syndrome usually presents in the first or second decade of life.^3^ Dermatologists should consider the diagnosis of H syndrome by recognizing its hallmark features, notably the presence of bilateral, symmetrical hyperpigmented indurated patches with overlying hypertrichosis, predominantly involving the medial aspects of the thighs. A recent report noted premature canities,^4^ and a case has been described with an adult onset of cutaneous manifestations only.^4^ Histopathology demonstrates widespread fibrosis and thickened collagen bundles.^5^ Histiocytes may be CD68 (+), S100 (+), and CD1a (−), and demonstrate emperipolesis, as appreciated in Rosai‐Dorfman disease.^6^

According to Low et al., “Treatments reported in the literature included systemic corticosteroids, methotrexate, cyclophosphamide, cyclosporine, 6‐mercaptopurine, interferon‐alpha, colchicine, anakinra, canakinumab, adalimumab, nonsteroidal anti‐inflammatory drugs, tocilizumab, and radiotherapy. These treatment regimens nevertheless were either ineffective or only resulted in partial responses.”.^6^

## 2 CASE REPORT

A case of a 12‐year‐old Iraqi female patient who consulted the Rheumatology Unit for joints contracture affecting both hands. She was born to first‐degree cousins with a low birthweight, delayed developmental milestones (she started walking at the age of 2.5 years), and protruding eyes since birth. She had a history of recurrent intermittent fever associated with sweating, fatigue, and generalized body aches.

At the age of 2, she was diagnosed with beta thalassemia minor and continued to take folic acid. At the age of four, she suffered from bilateral knee joint arthritis which was misdiagnosed as juvenile idiopathic arthritis. She was prescribed Methotrexate (MTX), but developed severe anemia as a side effect. MTX was eventually discontinued. She subsequently developed painless flexion deformities in all of her fingers, with the exception of her thumbs. In the same year, she was diagnosed with sensorineural deafness and subsequently received a hearing aid. At the age of five, she also underwent tonsillectomy surgery due to recurrent tonsillitis.

At the age of eight, she developed a hyperpigmented, tender rash on the inner aspects of her thighs and legs, associated with hypertrichosis at the same sites. Without prior trauma, pain, or swelling, painless foot deformities with laterally angled heels and overlapping toes, as well as medial deviation of the first metatarsal and lateral deviation of the big toe, were developed.

At the age of 10, she was prescribed antithyroid medication for 18 months after being diagnosed with hyperthyroidism. After six months, she gradually began to experience right hypochondriac abdominal pain, and an ultrasound revealed splenomegaly. The family declined the doctor's recommendation that a splenectomy be performed.

She was diagnosed with diabetes and started on insulin a year later; she was also diagnosed with celiac disease due to recurrent episodes of diarrhea associated with bloating and gas, and she is now on a gluten‐free diet. She presented to the rheumatology department at the time to assess her fingers' deformities.

**FIGURE 1:**
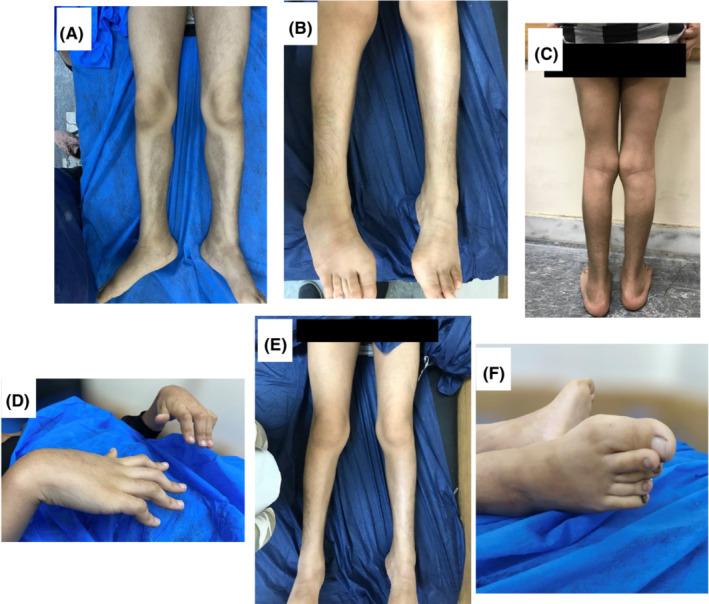
Those photographs for the hypertrichosis, genu valgus, and hallux valgus of the patients before treatment. Only images E is for the patient after the treatment.

**FIGURE 2:**
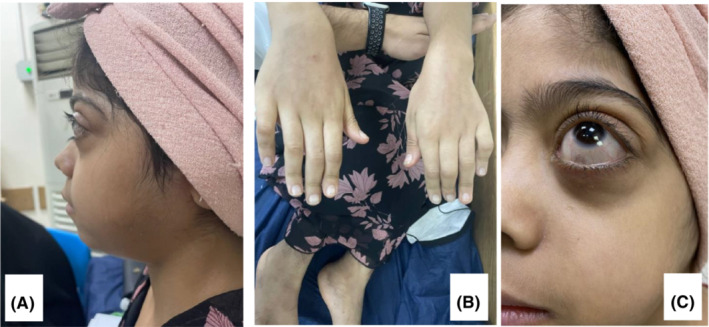
Hand deformities and exophthalmos of the eyes, dilated vessels and the arcus.

Her clinical examination revealed swollen cheeks and protruding eyes with corneal arcus and dilated scleral vessels (Figure 2A,C). She was malnourished and short‐statured, with a weight of 25 kg, a height of 135 cm, and her body mass index was 13.7 kg/m^2^. She had multiple palpable, freely mobile lymph nodes in the cervical, axillary, and inguinal regions. There were hyperpigmented tender skin lesions with hypertrichosis on the medial aspects of both thighs and the posteromedial aspects of both legs (Figure 1A–E). She had bilateral hallux valgus with irreducible flexion deformity of the hands and feet joints (Figures 1D,F and 2B). In addition to a pansystolic murmur over the mitral area, radiated to the axilla with no thrill or heave.

Her blood tests revealed hypochromic microcytic anemia (Hb 8.6 g/dl, MCV 49.7 fl), acute phase reactants were elevated [erythrocyte sedimentation rate: 77 mm/h (normal, 0–20)], [C‐reactive protein without titer: 22 mg/dl (normal, 0–5)]. normal renal, liver, and thyroid function tests. The celiac auto‐antibody screen was positive for Tissue Transglutaminase IgA antibody (tTG‐IgA, 45 IU/ml), Tissue Transglutaminase IgG (tTG‐IgA, 26.7 IU/ml), and anti‐gliadin IgG, 20.5 IU/ml (normal less than 12 IU/ml). IL‐6 was recorded as 17.56 pg/ml (normal, 0–7).

**FIGURE 3:**
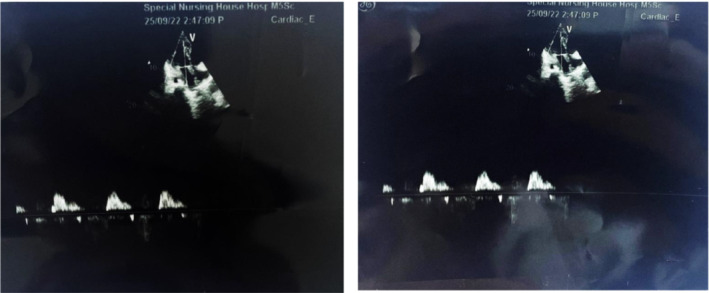
Echocardiography of the patient.

Echocardiography revealed mitral regurgitation and pulmonary stenosis. A neck ultrasound revealed multiple enlarged submandibular cervical LNs on the left side, with the largest measuring 13 mm and having an oval shape with calcification on the inside. A skin biopsy was performed after that, and the result was microscopic hyperpigmentation in the basal layer, sparse lymphoplasmacytic perivascular cells in the top layer of the dermis, and mild fibrosis (Figure 3).

**FIGURE 4:**
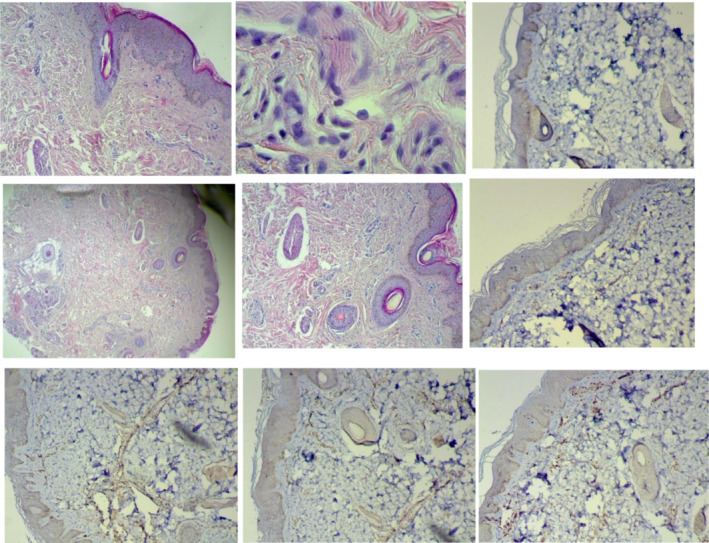
Histological pictures for the patient's biopsy, the upper six are related to CD20 and the lower three images are related to CD3.

In terms of the immunohistochemistry of highly light‐reactive CD3 and CD20 T‐ and B‐cell lymphocytes (as shown in Figure 4).

After that, in August 2021, the diagnosis was made by presenting the diagnostic criteria (hyperpigmentation, hypertrichosis, hyperglycemia, hallux valgus, hearing loss, heart problems (MR, PS), short stature with flexion deformities in combination with lymphadenopathy). She was prescribed a short course of oral prednisolone 10 mg/day that was rapidly tapered due to poor blood sugar control despite the use insulin with local tacrolimus ointment 0.1% that was suggested by a dermatology department with a sluggish response. Therefore, we prescribed her intravenous tocilizumab at a dose of 12 mg/kg every 2 weeks for 3 months. After one month, skin tenderness disappeared with normalization of CRP, IL6, and a drop in her ESR to 7 mm/hr. Furthermore, her body weight increased to 32 kg, and her height became 139 cm after 4 months of treatment. She is scheduled for a monthly follow‐up to receive 8 mg/kg of tocilizumab.

## 3 DISCUSSION

The H syndrome is an autosomal recessive disorder caused by bi‐allelic mutations in the SLC29A3 gene that encodes ENT3 (equilibrative nucleoside transporter 3), a nucleoside transporter protein. The ENT3 protein is found in intracellular membranes, especially in lysosomal and mitochondrial membranes.^7^ Because of the vast biochemical roles of nucleoside molecules, any disruption in metabolism and trafficking of nucleosides could result in various abnormal phenotypes.^6^ The H syndrome is considered a histiocytic disorder (histiocytosis lymphadenopathy pus syndrome).^6^ Other histiocytic maladies described in the literature, such as familial Rosai‐Dorfman (Faisalabad histiocytosis), and pigmented histiocytosis with insulin‐dependent diabetes, are now suspected to be part of a spectrum of disorders involving mutations of SLC29A3.^5^

H syndrome is an extremely rarely reported entity, more so in the Indian subcontinent, with only 10 cases reported from India till now in the literature. We present this case to increase awareness of this extremely rare and unique entity.^6^

## 4 CONCLUSION

H syndrome is a very rare genetic disorder that is rarely reported internationally or discussed in the medical literature. This case report from Iraq is demonstrated to increase the awareness regarding this unique topic and encourage doctors and specialists to focus on treating these patients, especially in the developing countries.

## AUTHOR CONTRIBUTIONS

**Nabaa Ihsan Awadh:** Conceptualization; data curation; writing – review and editing. **Faiq I. Gorial:** Resources; supervision. **Ahmed Dheyaa Al‐Obaidi:** Conceptualization; data curation; supervision; writing – original draft. **Mustafa Najah Al‐Obaidi:** Investigation; writing – original draft. **Ali H. AlNoori:** Resources; software; validation; writing – original draft. **Wesal K. Aljanabi:** Investigation; resources; supervision.

## CONSENT

Written informed consent was obtained from the patient to publish this report in accordance with the journal's patient consent policy.

## Data Availability

Data will be available on reasonable request.

## References

[1] Molho‐Pessach V , Lerer I , Abeliovich D , et al. The H syndrome is caused by mutations in the nucleoside transporter hENT3. Am J Hum Genet. 2008;83(4):529‐534. 18940313 10.1016/j.ajhg.2008.09.013 PMC2561939

[2] Razmyar M , Rezaieyazdi Z , Tayebi Meibodi N , Fazel Z , Layegh P . H syndrome masquerade as rheumatologic disease. Int J Pediatr. 2018;6(7):7965‐7971.

[3] Al‐Hamdi KI , Ismael DK , Saadoon AQ . H syndrome with possible new phenotypes. JAAD Case Rep. 2019;5:355‐357. 31008166 10.1016/j.jdcr.2019.02.003 PMC6453830

[4] Wang X , Sun J . Skin‐limited H syndrome in a Chinese man. Australas J Dermatol. 2019;60:243‐245. 30671934 10.1111/ajd.12968

[5] Molho‐Pessach V , Ramot Y , Camille F , et al. H syndrome: the first 79 patients. J Acad Dermatol. 2014;70:80‐88. 10.1016/j.jaad.2013.09.019 24172204

[6] Low DE , Tang MM , Surana U , Lee JY , Pramano ZAD , Leong KF . H syndrome—the first report in Malaysia. Int J Dermatol. 2019;58:e190‐e193. 31192449 10.1111/ijd.14518

[7] Chow VJ , Tsetsos N , Poutoglidis A , Georgalas C . Quality of life in sinonasal tumors: an up‐to‐date review. Curr Opin Otolaryngol Head Neck Surg. 2022;30(1):46‐57. 34889851 10.1097/MOO.0000000000000774

